# Mobile Health Apps in OB-GYN-Embedded Psychiatric Care: Commentary

**DOI:** 10.2196/mhealth.7988

**Published:** 2017-10-06

**Authors:** Aydan Mehralizade, Shayna Schor, Chad M Coleman, Claire E Oppenheim, Christy A Denckla, Christina PC Borba, David C Henderson, James Wolff, Sarah Crane, Pamela Nettles-Gomez, Avik Pal, Snezana Milanovic

**Affiliations:** ^1^ Boston Medical Center Boston, MA United States; ^2^ Harvard TH Chan School of Public Health Harvard University Cambridge, MA United States; ^3^ Boston University School of Medicine Boston, MA United States; ^4^ Boston University School of Public Health Boston, MA United States; ^5^ CliniOps Fremont, CA United States

**Keywords:** mHealth, eHealth, embedded psychiatric clinic, postpartum depression, mental health, OB-GYN, global health, reproductive health

## Abstract

This paper explores the potential benefits of the use of mobile health (mHealth) apps in obstetrician-gynecologist (OB-GYN)-embedded psychiatric clinics in the United States. First, we highlight the increasing trend of integrating mental health care within the OB-GYN context. Second, we provide examples of successful uses of mHealth in the global health context and highlight the dearth of available research in the United States. Finally, we provide a summary of the shortcomings of currently available apps and describe the upcoming trial of a novel app currently underway at the Mother-Child Wellness Clinical and Research Center at Boston Medical Center.

## Mental Health Care in the OB-GYN Setting

### Overview

Women are particularly vulnerable to increased mental health problems in the peripartum and antepartum periods, and again at menopause [1]. Unfortunately, these symptoms of mental illness go largely undiagnosed and untreated because of time constraints and lack of trained providers [2,3]. Obstetrician-gynecologists (OB-GYNs) are often the only regular health care providers for women. OB-GYNs see a full third of all nonillness-related (ie, prophylactic) visits for women under 65 [4]. Among women with mental illness, the highest rates of depression occur in women of lower socioeconomic status, minority women, and immigrant women. These women tend to seek primary care within OB-GYN settings [5].

For the abovementioned reasons, health care professionals have been advocating for an integrated care approach that incorporates psychiatric care into the OB-GYN setting [6,7]. Available evidence from randomized trials suggests that integrated care has a significant positive impact on depression outcomes, especially among women of lower socioeconomic status [8]. In addition, researchers have estimated that mental health care integrated into the primary care setting can save up to US $48 billion annually in the United States [9].

### mHealth Care in the Global OB-GYN Context

A key emerging technology that could potentially advance integrated care is mobile health (mHealth), particularly mHealth apps. mHealth is defined as “medical and public health practices supported by mobile devices including mobile phones, patient monitoring devices, personal digital assistants, and other wireless devices” [10]. mHealth sits at the intersection of electronic health and mobile phone technology [11]. Mobile phones are the most popular mobile technology used today and their use will only continue to increase. According to estimates by the mobile communications giant Ericsson, by 2019, there will be 9.3 billion mobile subscriptions in the world; 5.6 billion of these will be mobile phone subscriptions [12]. Mobile phones have a clear advantage over many other technologies due to their portability, Internet connectivity, and power to run several apps at once. Tablets connected to the Internet by Wi-Fi, such as the Apple iPad and Google Nexus, are increasingly used in the health field to deliver educational material for patients and medical professionals, run medical algorithms, and provide access to patient data [13].

A mobile app is software that runs on a mobile device [14]. Between 2005 and 2011, the number of mHealth apps launched increased by 30% [15]. As of 2015, there were over 40,000 medical apps available for tablets and mobile phones and over 247 million people had downloaded an mHealth app [16]. mHealth apps provide appointment tracking, mood/behavior tracking, community engagement, patient education, and support for behavior change [17].

A recent meta-analysis found that mHealth apps in the OB-GYN setting have been shown to improve women’s health outcomes in the global health context (eg, increased antenatal care attendance and improved breastfeeding practices) [18]. For example, the Mobile Alliance for Maternal Action (MAMA) project provides information to pregnant women based on the woman’s estimated date of delivery. MAMA was a 3-year, US $10 million investment cofunded by the United States Agency for International Development, the mHealth Alliance, and the United Nations Foundation, among others, with a goal of delivering health messaging via mobile phones to expectant mothers in Bangladesh, India, and South Africa. MAMA successfully achieved its goals of promoting institutional deliveries, antenatal and postnatal care visit uptake, and exclusive breastfeeding in the three original locations. As evidence of the project’s ability to scale up, in 2014, the global coordination office of MAMA closed, and in-country partners took full ownership of their respective projects. Currently, the MAMA model is being used in 54 countries by 161 organizations [19]. In a relatively new field of mHealth, where successful pilot studies struggle to maintain funding, MAMA is a leading example of an effective and sustainable intervention.

The global health literature contains many more examples of successful use of mHealth in women’s primary health care settings. In Oro State, Nigeria, a successful mHealth project improved antenatal care appointment adherence and increased diagnosis and treatment of pregnancy complications. This was achieved by giving pregnant women mobile phones and sending them weekly short message service (SMS) text messages with information on potential complications and other educational materials [20]. In Rwanda and Zanzibar, mHealth is reported to have improved maternal health outcomes and reduced neonatal mortality [21,22].

### mHealth Care in the OB-GYN Setting in the United States

Despite evidence that the use of mHealth apps in global OB-GYN settings results in improved outcomes, in the United States there is scant evidence on the use of mHealth to improve health outcomes [23]. A study in 2012 found that 59% of patients in emerging markets use at least one mHealth app compared to 35% of patients in the developed world [14]. This gap represents an opportunity for integrated psychiatric services within OB-GYN settings to incorporate mHealth technology, capitalize on the experience gained in the global health field in the past 10 years, and improve mental and reproductive health outcomes of their patients.

## The Mother-Child Wellness Clinical and Research Center

### Overview

**Figure 1:**
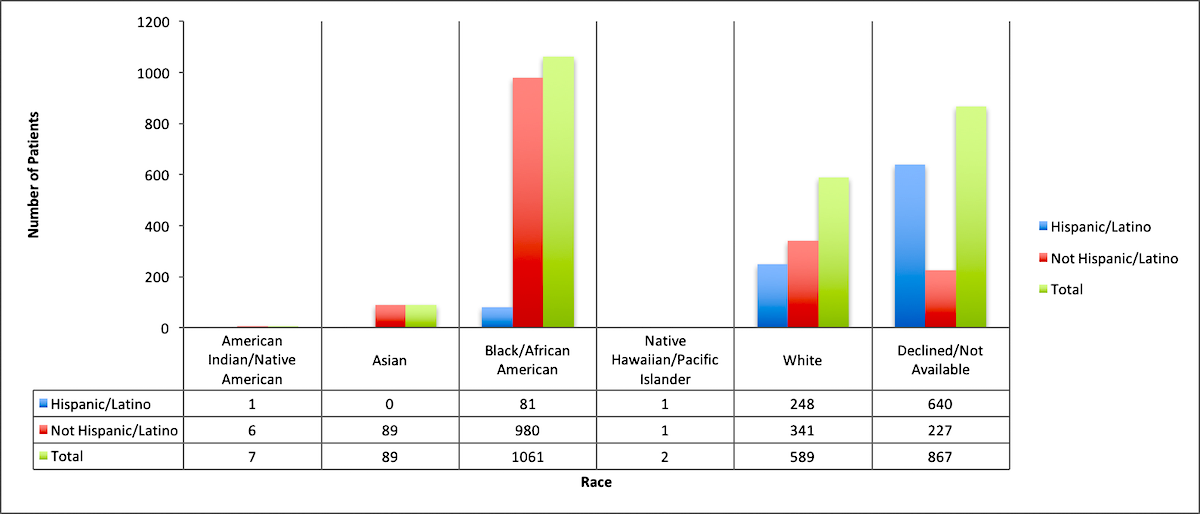
Race distribution of the 2615 women who delivered at Boston Medical Center between May 1, 2015 and May 1, 2016.

**Figure 2:**
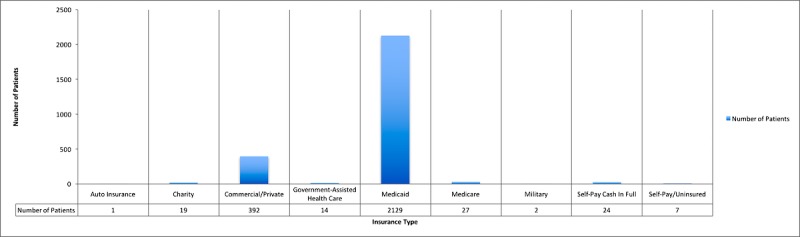
Health insurance distribution of the 2615 women who delivered at Boston Medical Center between May 1, 2015, and May 1, 2016.

The Mother-Child Wellness Clinical and Research Center (MCWCRC) at Boston Medical Center, founded in 2016, is pursuing this strategy to enhance care for women and children by integrating psychiatric care in the OB-GYN setting. The patient population of Boston Medical Center is diverse in terms of socioeconomic status and ethnic and racial background: 71.52% are nonwhite, 33.3% are Hispanic, and 82.49% are Medicaid patients (detailed demographics of the MCWCRC patient population are provided in Figures 1 and 2). Women who fit into these demographic categories are more likely to report institutional and stigma-related barriers to accessing mental health care when visiting an OB-GYN clinic, despite high interest in receiving some form of psychiatric care [24]. In fact, the no-show rate at the MCWCRC is approximately 50% and patients identify lack of transportation options as a major barrier to accessing care (unpublished internal research).

In order to improve the quality of services offered to its target population, the MCWCRC plans to design and integrate an mHealth app into its continuum of care. The proposed mHealth app will be used to conduct depression screening, reduce loss to follow-up, integrate patients’ mental health records with the clinic’s existing electronic medical records system (ie, Epic), help patients track their appointments, record behavioral symptoms, provide educational information, engage with the community, and alert providers to patients who may need a mental health intervention.

The MCWCRC at Boston Medical Center decided to build a customized mHealth app because the mHealth apps available on the US market do not meet all the needs for an integrated psychiatric facility embedded within an OB-GYN clinic. Apps that track mood and help patients cope with their psychiatric conditions do not provide other essential functions, such as appointment tracking, community engagement, or prenatal and postnatal education. On the other hand, apps aimed specifically at prenatal and postnatal education, appointment tracking, and community engagement lack the crucial aspect of mental health engagement. A brief summary of various apps available on the market is provided in Table 1 below. No information on the number of subscribers to each app can be found in the published literature.

### mHealth Trial at the Mother-Child Wellness Clinical and Research Center

The MCWCRC team, working with CliniOps, a vendor specializing in e-solutions for monitoring and patient management, is implementing a custom-built tablet and mobile phone app at Boston Medical Center [25]. CliniOps has already developed and implemented a mental health-focused app in the global health context and is currently in the process of customizing the app in accordance with the specific needs of the MCWCRC population. The prospective cohort study will take place in two phases: (1) a limited 3-month trial (ie, pilot phase or Phase I), slated to begin in the fall of 2017, and (2) a subsequent clinic-wide trial (ie, Phase II) scheduled to last 12 months.

**Table 1:** Comparison of prenatal and pregnancy apps available on the market in terms of various functions.

App	US or global	Appointment tracking	Patient education	Community engagement	Behavior/mood tracking	Payment
BabyCenter	Global^a^	X	X	X		In-app purchases
Glow Nurture	Global^a^	X	X	X		In-app purchases
Text4baby	US	X	X			Free
myStrength	US				X	Free
PPD ACT^b^	US				X	Free
MAMA^c^	Global^d^	X	X			Free

^a^Apps that we mostly used in developed English-speaking countries (ie, United States, United Kingdom, Australia, and New Zealand).

^b^PPD ACT: Postpartum Depression: Action Towards Causes and Treatment.

^c^MAMA: Mobile Alliance for Maternal Action.

^d^Apps used in developing countries.

In the pilot phase (Phase I), all pregnant and postpartum patients entering the clinic will be asked to complete an iPad-administered questionnaire, available in both English and Spanish. We anticipate at least 1000 patients will be enrolled in the pilot phase of the study. The iPad questionnaire will collect data on demographics and general psychiatric symptoms. Namely, it will use the following psychiatric scales: Edinburgh Postnatal Depression Scale (EPDS), the Life Events Checklist for the Diagnostic and Statistical Manual of Mental Disorders, Fifth Edition (LEC-5) Trauma Scale, and the Andrew Cherry-Oklahoma Co-Occurring Disorders (AC-OK COD) Screen [26-28]. The EPDS is a commonly used, self-screening tool used to identify depression. The LEC-5 Trauma Scale is a self-screening measure meant to identify traumatic events in the respondent’s life. The AC-OK COD Screen is a self-administered tool to establish whether the respondent experiences co-occurring mental illness and substance abuse. The contents of the questionnaire and the scales will be read aloud to patients with low literacy levels. If a patient exceeds the EPDS threshold for depression (>10), an automatic educational module will be triggered regarding depression in pregnancy and postpartum. In addition, a care choice menu will be activated asking the patient whether she would like to contact her medical doctor or a case manager, or if she would like to access individual or group therapy sessions. Subsequently, patients will be offered the option of consulting a mental health professional on-site. Furthermore, patients will be asked for their consent to participate in a future research study, as well as whether they would be receptive to downloading a mobile phone app for that purpose. The mobile phone app by CliniOps will provide educational information on gestation and mental health, as well as opportunities for community engagement, appointment reminders, and mood/behavior tracking. After the conclusion of the pilot study, the data will be analyzed, and the respondents’ acceptance levels of the mobile phone app will be measured.

During the clinic-wide trial (Phase II), patients will choose between downloading the mobile phone app or receiving follow-up care as usual. The clinic-wide phase will thus be an open-label study; the differences in treatment adherence (ie, reflected in the no-show rate), clinical outcomes (ie, time to depression remission), and health care utilization between the two groups (ie, app users and standard care users) will be measured.

**Figure 3:**
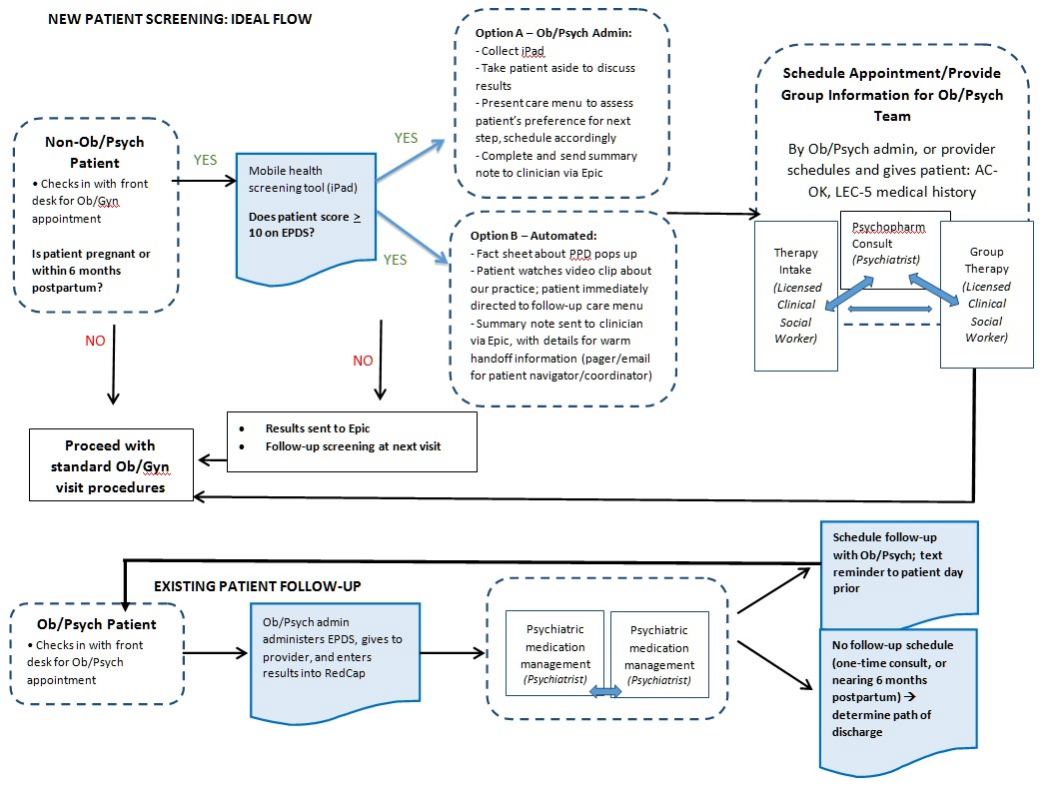
Visual representation of the patient flow during the pilot phase of the mHealth prospective cohort study in the Mother-Child Wellness Clinical and Research Center at Boston Medical Center. AC-OK: Andrew Cherry-Oklahoma Co-Occurring Disorders Screen; EPDS: Edinburgh Postnatal Depression Scale; LEC-5: Life Events Checklist for the Diagnostic and Statistical Manual of Mental Disorders, Fifth Edition; Ob: obstetric; Psych: psychiatric; Ob/Gyn: obstetrician-gynecologist; PPD: postpartum depression.

The long-term goal of this mHealth trial (Phase II) is to incorporate the patients’ questionnaire data into their Epic record and send screening summary alerts to their respective obstetricians and mental health providers. Figure 3 provides a visual representation of the patient flow during the pilot phase of the trial.

The use of mHealth apps among patient populations should be carefully considered in the context of the socioeconomic setting. This concern is due to the expenses associated with maintaining sufficient data to receive SMS text messages and read educational materials online. In the United States, 68% of adults are estimated to have a mobile phone, while 45% have a tablet [29]. The socioeconomic composition of the patient population must be taken into account when designing a mobile health intervention to ensure equity and reliability of the trial. In addition, patients’ educational levels must also be considered. The MCWCRC team in conjunction with CliniOps is cognizant of the literacy level concerns and is committed to making the tablet- and mobile phone-based app accessible to the Boston Medical Center patient population. There is encouraging evidence on this topic in the published literature: people with low self-reported health literacy are likely to seek information from mobile phone-based health apps [30].

In addition, global health experience shows that the uptake of successful pilot studies is often limited [31]. With some rare exceptions, such as the MAMA project, mHealth interventions often fail to scale up and demonstrate long-term utility. The MCWCRC at Boston Medical Center will seek to overcome this trend by showing that a preventative, embedded psychiatric approach will lead to cost savings in the long term, not in the least part by improving the current 50% no-show rate, capturing patients with nascent symptoms early on, and increasing utilization of clinic services by providing screening and education on the spot. We anticipate that the use of our mobile platform, which includes the iPad behavioral health screen and the mobile phone app aimed at education and care coordination, will prevent patients from seeking help in the emergency department for conditions that can be successfully addressed within the embedded psychiatric clinic; in the long run, this could lead to cost savings for the medical center, the taxpayer, and the patient.

## Conclusions

Prior studies have shown that mHealth apps have the potential to improve outcomes among patients in OB-GYN settings; further research is needed to evaluate whether these benefits extend to obstetric clinics with embedded psychiatric care. The wealth of information available from global health research indicates a high potential for improved mental and reproductive health outcomes. However, limited research has been conducted in the United States to determine the health outcome benefits associated with mHealth, particularly in a low socioeconomic status, inner city population. Furthermore, there is no comprehensive app on the US market that incorporates patient education, behavior change, mood tracking, appointment reminders, and community engagement. The MCWCRC at Boston Medical Center will endeavor to fill this gap with the upcoming mHealth app trial.

## Abbreviations

AC-OK COD: Andrew Cherry-Oklahoma Co-Occurring Disorders
EPDS: Edinburgh Postnatal Depression Scale
LEC-5: Life Events Checklist for the Diagnostic and Statistical Manual of Mental Disorders, Fifth Edition
MAMA: Mobile Alliance for Maternal Action
MCWCRC: Mother-Child Wellness Clinical and Research Center
mHealth: mobile health
OB-GYN: obstetrician-gynecologist
PPD ACT: Postpartum Depression: Action Towards Causes and Treatment
SMS: short message service
